# An Improved Detection and Quantification Method for the Coral Pathogen *Vibrio coralliilyticus*

**DOI:** 10.1371/journal.pone.0081800

**Published:** 2013-12-10

**Authors:** Bryan Wilson, Andrew Muirhead, Monika Bazanella, Carla Huete-Stauffer, Luigi Vezzulli, David G. Bourne

**Affiliations:** 1 Centre for Marine Microbiology and Genetics, Australian Institute of Marine Science (AIMS), Townsville, Queensland, Australia; 2 Fachhochschule Krems, Krems, Austria; 3 Università degli Studi di Genova, Genoa, Italy; Universidade Federal do Rio de Janeiro, Brazil

## Abstract

DNA- and RNA-based PCR and reverse-transcription real-time PCR assays were developed for diagnostic detection of the *vcpA* zinc-metalloprotease implicated in the virulence of the coral pathogen *Vibrio coralliilyticus*. Both PCR methods were highly specific for *V. coralliilyticus* and failed to amplify strains of closely-related *Vibrio* species. The assays correctly detected all globally occurring *V. coralliilyticus* isolates including a newly-described isolate [TAV24] infecting gorgonians in the Mediterranean Sea and highlighted those isolates that had been potentially misidentified, in particular *V. tubiashii* strains ATCC 19105 and RE22, historically described as important oyster pathogens. The real-time assay is sensitive, detecting 10 gene copies and the relationships between gene copy number and cycle threshold (C*_T_*) were highly linear (R^2^≥99.7). The real-time assay was also not affected by interference from non-target DNA. These assays are useful for rapid detection of *V. coralliilyticus* and monitoring of virulence levels in environmental samples, allowing for implementation of timely management steps to limit and possibly prevent losses due to *V. coralliilyticus* infection, as well as furthering investigations of factors affecting pathogenesis of this important marine pathogen.

## Introduction

Coral reefs represent one of the most biologically diverse ecosystems in the world, as well as playing a vital role in supporting local communities by way of coastal protection, food production and tourism [1]–[3]. In recent decades however, coral reef health has been increasingly compromised by natural and anthropogenic disturbances [2] and reports of disease outbreaks have escalated [4]. Bacterial pathogens have been identified as causative agents for a number of these diseases [3] and in particular, members of the *Vibrionaceae* family [5]–[7] implicated in outbreaks. Of these, *V. coralliilyticus* is amongst the best studied [5], [8]–[21] and investigations into strains isolated from white syndrome disease outbreaks in the Indo-Pacific [6] revealed the expression of a zinc-metalloprotease demonstrated to cause coral tissue damage [15].

Zinc-metalloproteases are common in prokaryotic organisms, having an essential role in maintenance of cellular homeostasis. In pathogenic organisms (particularly opportunists), the action of these proteases enhances vascular permeability, necrotic tissue damage and cytotoxicity, therefore facilitating bacterial invasion [22] and the infection process. Expression of the metalloproteases in *Vibrio* species is growth-phase dependent and positively regulated by various quorum-sensing systems [23]–[27]. The zinc-metalloprotease of *V. coralliilyticus* has been shown to cause coral tissue lesions [8], [15] concurrent with white syndrome aetiology and so detection of this important virulence factor could provide early indications of infection and assist agencies in developing strategies to effectively contain coral disease outbreaks. There is a pressing need for disease diagnostics in coral science [28], as the principal method for coral disease detection currently involves field-based observations of gross macroscopic symptoms which, whilst requiring anatomical knowledge for correct diagnosis [29], also detect only the latest stages of disease, by which time remedial management measures are ineffective. PCR-based diagnostic methods are rapid, specific and sensitive with the detection of *Vibrio* species by these means previously validated in other systems [17], [30]–[34]. A real-time assay to detect *V. coralliilyticus* previously published targeted the *dnaJ* gene using the TaqMan qPCR system for amplification from cells and genomic DNA [17]. In this study, we describe the development of qualititative PCR and quantitative reverse transcription real-time PCR (using SYBR Green I dye) assays with an improved capacity not only for strain classification but also for quantification of viable and active *V. coralliilyticus* populations.

## Results

### Standard curves and detection range

Standard curves constructed from serial tenfold dilutions of known concentrations (10^0^–10^7^ copies) of the pCR2.1-TOPO plasmid carrying the *vcpA* gene were successfully generated and allowed quantitative detection of the *vcpA* gene using both developed primer sets (vcpAF-vcpAR and vcpARTF-vcpARTR). Standard curves for both real-time PCR and PCR showed high linear correlation, with linear regression coefficients (R^2^ value) equal to or greater than 0.997 and both primer sets were able to detect the *vcpA* gene down to levels of 10 gene copies.

### Evaluation of primers

*In silico* comparisons of the primer pairs against sequences stored in the NCBI database using BLASTn [35] revealed that both VcpAF and VcpARTF forward primers matched the *V. tubiashii* zinc-metalloprotease *vtpA* (Accession Numbers EU675309 and FJ455119-FJ455121) though neither of the reverse primers (VcpAR and VcpARTR) matched any non-target species. Using a standard PCR approach with the primer set VcpAF and VcpAR, a single amplicon (1.8 kB) was observed when DNA from all globally-occurring *V. coralliilyticus* strains (Table 1) was added to single reactions, the exception being strain P3 (LMG 23695) which did not amplify. No amplicons were observed when other *Vibrio* species were tested (Table 1), including *V. tubiashii* ATCC 19109*^T^*. The amplicons obtained for the *vcpA* genes were verified by sequencing and confirmed the specificity of this assay for *V. coralliilyticus* strains. The same strain specificity was observed using the real-time PCR primer set (VcpARTF and VcpARTR) in standard PCR, with only one size of amplicon (166 bp) obtained for *V. coralliilyticus* isolates, with a mean melting temperature of 83.41°C±0.15. For products from isolates BH6, C1, C2 and TAV24, the melt curve analysis revealed a different peak corresponding to a melting temperature of 84.34°C±0.11. Using these same primers in reverse transcription real-time PCR with cDNA from *V. coralliilyticus* strains, C*_T_* values of 8–14 cycles were obtained, whilst other non-*V. coralliilyticus* species were detected at C*_T_* values of 21–31 cycles (Table 1). The RT-PCR result for *V. coralliilyticus* strain P3 (C*_T_* value of 25.67±0.58) confirmed the result observed in the standard PCR approach. The C*_T_* values for the no template control (NTC) were between 28–33 cycles.

**Table 1:** Species and strains tested.

Species	Isolate (Strainα)	Reference	C*_T_*±SD
*Vibrio coralliilyticus*	P1 (LMG 23696)	[6]	9.54±0.42
	P2 (LMG 23691)	[6]	8.95±0.77
	P3 (LMG 23695)	[6]	25.67±0.58
	P4 (LMG 23693)	[6]	8.85±0.76
	P5 (LMG 23692)	[6]	10.81±0.43
	P6 (LMG 23694)	[6]	8.54±0.42
	BH1 (LMG 20984*^T^*)	[5]	8.52±0.34
	BH2 (LMG 21348)	[8]	9.28±0.39
	BH3 (LMG 21349)	[8]	13.46±0.33
	BH4 (LMG 21350)	[8]	9.30±0.73
	BH5 (LMG 10953)	[8]	8.19±0.44
	BH6 (LMG 20538)	[8]	9.01±0.35
	C1 (PaD1.44)	[19]	9.41±0.84
	C2 (PaD1.51)	[19]	8.36±0.23
	TAV24	[61]	8.21±0.62
*AliiVibrio fischeri*	DSM 507		30.54±1.83
*Vibrio alginolyticus*	ATCC 17749		25.76±0.14
*Vibrio brasiliensis*	DSM 17184		23.78±0.16
*Vibrio calviensis*	DSM 14347		24.33±0.24
*Vibrio campbellii*	ATCC 25920		29.46±1.49
*Vibrio fortis*	DSM 19133		25.61±0.12
*Vibrio furnissii*	DSM 19622		29.65±1.62
*Vibrio harveyi*	DSM 19623		30.22±0.95
*Vibrio natriegens*	ATCC 14048		26.51±0.04
*Vibrio neptunius*	LMG 20536		27.64±0.45
*Vibrio ordalli*	ATCC 33509		21.68±0.17
*Vibrio parahaemolyticus*	DSM 10027		27.75±1.42
*Vibrio proteolyticus*	ATCC 15338		27.41±0.08
*Vibrio rotiferianus*	DSM 17186		30.45±0.92
*Vibrio splendidus*	DSM 19640		28.94±1.49
*Vibrio tubiashii*	ATCC 19109*^T^*		26.55±0.11
	RE22	[36]	8.91±0.05
*Vibrio xuii*	DSM 17185		28.42±0.97
*Escherichia coli*	DH5α		30.41±1.27

α Strain designations beginning LMG were from the Belgian Coordinated Collections of Microorganisms, ATCC were from the American Type Culture Collection, DSM were from the Deutsche Sammlung von Mikroorganismen und Zellculturen GmBH, C1 and C2 were provided by Pamela Morris, Hollings Marine Laboratory, USA and *V. tubiashii* RE22 was provided by Claudia Häse, Oregon State University, USA.

**Figure 1:**
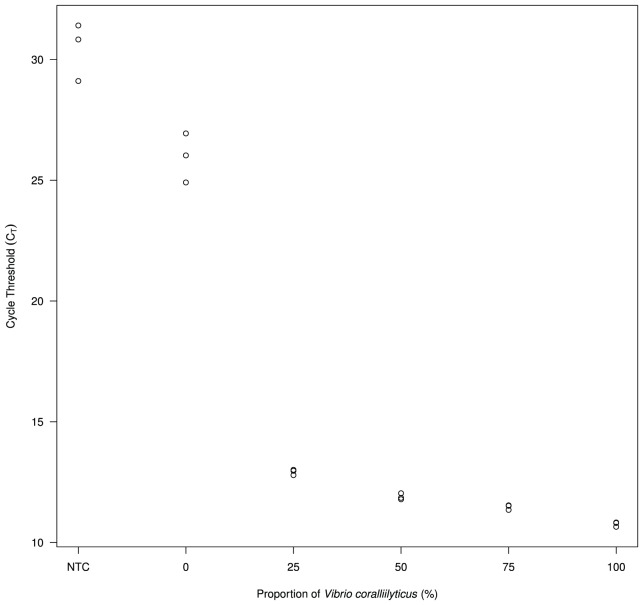
Real-time PCR detection of *V. coralliilyticus* P1 cells in mixed cultures containing proportional numbers of *V. tubiashii* 19109*^T^* cells. Each mixed culture was sampled in triplicate.

The *vcpA* real-time PCR (VcpARTF-VcpARTR) specifically detected only *V. coralliilyticus* strains when a non-target culture of *V. tubiashii* ATCC 19109*^T^* was mixed in defined ratios (0∶100, 25∶75, 50∶50, 75∶25 and 100∶0%, respectively) after both target and non-target cultures were grown overnight and normalised to an OD_600_ of 1.0 (approximately 8×10^8^ cells) and total DNA subsequently extracted and amplified (Fig. 1). The target *V. coralliilyticus* strains were detected at similar C*_T_* values (10.65–13.01) to those observed previously (Table 1) and C*_T_* values were positively correlated with *V. coralliilyticus* proportions (R^2^ = 0.956), indicating that non-target *V. tubiashii* did not interfere with *vcpA* gene amplification. Both 100% *V. tubiashii* and NTC C*_T_* values (25.96±1.02 and 30.45±1.20, respectively) were also within expected ranges.

The specificity of the *vcpA* primers was validated by amplification of the same target and non-target genomic DNA mixtures using real-time PCR primers for the *V. tubiashii* zinc-metalloprotease *vtpA* [33]. These primers matched the *V. coralliilyticus vcpA* sequence *in silico* and so we intended to demonstrate that whilst the *vcpA* primers could detect changes in *V. coralliilyticus* concentration in the template mixture, the *vtpA* primers would amplify both the target and *V. tubiashii* non-target templates and so C*_T_* values would be similar for all mixtures. However, when the template mixtures were amplified by real-time PCR using the *vtpA* forward and reverse primers, we obtained the same result as for the *vcpA* primers; namely that C*_T_* values were positively correlated with *V. coralliilyticus* template DNA concentrations whilst 100% *V. tubiashii* ATCC 19109*^T^* and NTC C*_T_* values were again similar (data not shown).

**Figure 2:**
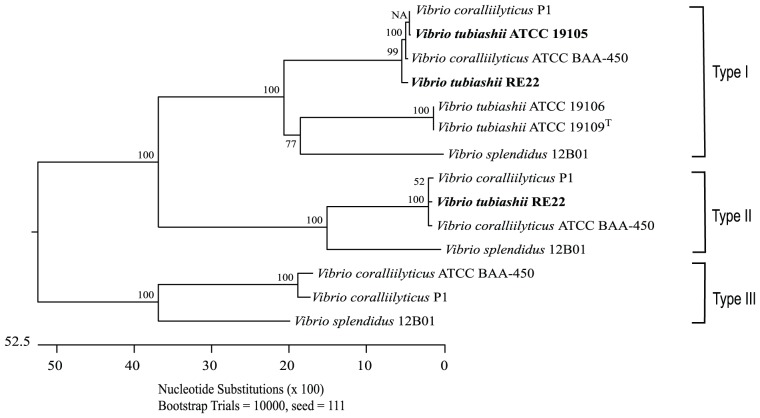
Phylogenetic tree (using the neighbour-joining method) of Type I (1.8 kb), II (2.3 kb) and III (2 kb) metalloprotease genes from *V. coralliilyticus* and *V. tubiashii* (with *V. splendidus* included as an outgroup). Figures at nodes indicate bootstrap values for 10000 bootstrap repetitions.

Gharaibeh *et al.* (2009) developed the *vtpA* primers using the closely related *V. tubiashii* strains RE22 and ATCC 19105 [36]. Real-Time PCR of RE22 using both *vtpA* and *vcpA* primers resulted in C*_T_* values similar to those for *V. coralliilyticus* P1, at 10.96±0.16 and 8.91±0.05, respectively. Closer examination of nucleotide sequence data for all published metalloprotease genes for *V. coralliilyticus* (including sequences from two genomes, P1 and BAA-450) and *V. tubiashii* (with sequences from the genomes for strains ATCC 19109*^T^* and 19106) (Fig. 2) revealed significant differences between the *V. tubiashii* strains; as described previously [20], *V. coralliilyticus* has three copies of the metalloprotease (defined here as Types I, II and III) whereas *V. tubiashii* strains ATCC 19106 and 19109*^T^* have only one copy (Type I). *V. tubiashii* strain ATCC 19105 has one published Type I metalloprotease (*VtpA*) whilst RE22 however has two published metalloprotease gene copies (*vtpA* and *vtpB*) [37] grouped within Types I and II, respectively, and these are more closely related to metalloproteases from *V. coralliilyticus* strains than *V. tubiashii* strains ATCC 19109*^T^* and ATCC 19106 (Fig. 2). Recently, the unannotated *de novo* assembly of the genome of *V. tubiashii* RE22 has been published online http://figshare.com/articles/Contigs_de_novo_RE22/90805(http://figshare.com/articles/Contigs_de_novo_RE22/90805). Sequence comparisons of eight housekeeping gene loci (*ftsZ*, *gapA*, *gyrB*, *mreB*, *pyrH*, *recA*, *rpoA* and *topA*) from *V. coralliilyticus* BAA-450 and *V. tubiashii* strains ATCC 19106 and 19109*^T^* with the RE22 assembly revealed that the mean identity of matches with BAA-450 sequences was 98%, whilst with sequences from strains ATCC 19106 and 19109*^T^*, it was 88%. In a search of the RE22 assembly for the *V. coralliilyticus* BAA-450 Type III metalloprotease (Accession No. ZP_05885140.1), we also discovered a contig (RE22_contig_1616) containing a 1553 bp region with a 98% sequence identity to this gene.

## Discussion

The *Vibrionaceae* are abundant in aquatic environments and are often found in high densities in association with marine organisms [38]–[40]. Historically, these opportunistic pathogens have long been associated with diseases of animals reared for aquaculture [41]–[45] but recently, the evidence for their increasingly important role in coral disease has been growing [2], [3] and a number of putative candidates have been studied, including *V. shilonii* [39], [46]–[50], *V. harveyi* [51], [52] and *V. coralliilyticus* [5], [8]–[21]. The relationship of the metalloproteases to pathogenicity amongst the prokaryotes is now well accepted [22] and their efficacy as virulence factors has been studied in a number of *Vibrio* species including *V. tubiashii* [37], [53], *V. splendidus* [54], [55], *V. vulnificus* [56]–[59] and *V. anguillarum* [60]. The zinc-metalloprotease of *V. coralliilyticus* has been shown to play an important role in the organism's virulence towards corals and their symbiotic zooxanthellae [8], [15], [20] and therefore this metalloprotease gene (*vcpA*) provided an attractive target for a detection assay. This study successfully developed specific primers to detect the *vcpA* gene in *V. coralliilyticus* using both PCR and reverse transcription real-time PCR approaches.

The PCR and real-time PCR primer sets designed in this study were able to specifically and routinely amplify DNA from all putative *V. coralliilyticus* isolates (Table 1), including a newly-described isolate (TAV24) infecting gorgonians in the Mediterranean Sea [61]. The specificity of the assays were such that they confirmed the misidentification of an isolate (P3) as *V. coralliilyticus* from a previous study [15] and also amplified an isolate (C2) which could not be detected by the real-time PCR assay of Pollock *et al.* (2010). Interestingly, melting curve analyses of the global *V. coralliilyticus* isolates revealed that the melting curve profiles for strains BH6, C1, C2 and TAV24 were different from all other *V. coralliilyticus* strains and this is correlated with the sequence divergence of their metalloproteases, as shown in a previous study [18]. When the GC content, which determines the melting curve profile, of the entire *vcpA* ORF nucleotide sequences derived from all global isolates was examined, a difference of >0.5% between strains BH6, C1, C2 and TAV24 (47.50±0.11%) and other *V. coralliilyticus* strains (46.90±0.16%) was observed. We are currently investigating the virulence of these two divergent clades of metalloproteases in *V. coralliilyticus* and melting curve profile analyses provide a rapid means for classification of these genes from newly sourced isolates.

A significant finding of the study was the serendipitous discovery of the shared metalloprotease and potential misclassification of *V. tubiashii* strains ATCC 19105 and RE22. The strain ATCC 19105 was first described by Tubiash *et al.* [62] as *V. anguillarum* and deposited in the American Type Culture Collection along with strains ATCC 19106 and ATCC 19109*^T^*. In a later study by Hada *et al.* [63], the three strains were reclassified as *V. tubiashii* using DNA-DNA hybridisation; interestingly, whilst ATCC 19106 shared a 96% DNA homology with ATCC 19109*^T^*, ATCC 19105 had only an 83% degree of reassociation with the type strain. Similarly, in comparative analyses of strains ATCC 19109*^T^* and ATCC 19105, there were notable differences in both the outer-membrane proteins produced and the expression of siderophores in iron utilisation [64], [65]. The historical confusion that surrounds identification of *V. coralliilyticus* and *V. tubiashii* species was first highlighted in the original article describing *V. coralliilyticus* [9], whereby the authors reclassified *V. tubiashii* LMG 10953 as *V. coralliilyticus* (strain BH5; Table 1). Following the incongruous quantitative PCR results obtained for *V. tubiashii* strains ATCC RE22 and 19109*^T^*, we constructed a phylogenetic tree using the metalloprotease sequence data for *V. coralliilyticus* and *V. tubiashii* strains (Fig. 2). *V. coralliilyticus* has three metalloproteases (Types I, II and III) [20], however, the recently published genomes for *V. tubiashii* strains ATCC 19106 [66] and 19109*^T^* (Accession Number NZ_AFWI00000000.1) identified each having only a single Type I metalloprotease. To date, the genome for ATCC 19105 has not been sequenced but two metalloproteases, *vtpA* [37] and *vtpB* [67] (Types I and II, respectively) have been characterised in the closely-related strain RE22 and these can be seen to cluster with those of *V. coralliilyticus*; the Type III metalloprotease has also been found within the unannotated RE22 genome recently published online http://figshare.com/articles/Contigs_de_novo_RE22/90805(http://figshare.com/articles/Contigs_de_novo_RE22/90805). The ATCC 19105 Type I and RE22 Type I and Type II metalloproteases shared >98% sequence identity with *V. coralliilyticus* strains ATCC BAA-450 and P1, compared with <72% for the Type I metalloproteases of *V. tubiashii* strains ATCC 19106 and 19109*^T^*. Whilst the presence of highly similar (>98%) multiple zinc-metalloprotease genes (and in the case of RE22, a number of other gene loci) strongly suggests a relatedness of ATCC 19105 and RE22 to *V. coralliilyticus*, further genomic sequence data is required to confirm their relationships and this is currently under investigation. Certainly, the virulence factors and mechanisms of pathogenesis studied independently in the shellfish pathogens RE22 and ATCC 19105 and coral pathogen *V. coralliilyticus* appear to be identical. Two isolates of *V. coralliilyticus* (BH5 and BH6) were previously obtained from diseased bivalve larvae (in the UK and Brazil, respectively) [9] but there is no evidence to confirm whether these strains were involved in shellfish pathogenesis. Since ATCC 19105 and RE22 have been shown to be major commercial pathogens of bivalves [37], if whole genome sequence data confirm their suspected identities, it would seem that the threat of *V. coralliilyticus* is no longer limited to coral reefs.

Due to the relative homogeneity of the 16S rRNA gene in *Vibrio* species, alternative gene targets for identification have been investigated and indeed the *dnaJ* gene has been suggested as a promising candidate [34], [68]. Pollock *et al.* (2010) previously designed a real-time PCR assay with TaqMan fluorescent probes for the *dnaJ* gene to detect *V. coralliilyticus* in environmental samples which was successful in specifically detecting twelve of thirteen tested *V. coralliilyticus* isolates. However, the assay developed during this current study improves upon this previous work due to its increased efficacy for discriminating *V. coralliilyticus* strains. The current assay was also optimised for analysis of mRNA, facilitating quantification of *in situ* gene expression in viable organisms, in contrast with genomic DNA which can be derived from extant or non-viable cells or even directly from seawater [69]. In their role of opportunist pathogens, *Vibrio* species are also known to comprise the normal €resident€ microbial communities associated with healthy corals [70] and so expression of a virulence factor such as the metalloprotease is useful in assessing the potential for a disease event. Indeed, zinc-metalloproteases have been targeted previously to monitor virulence during pathogenesis of oysters [33].

Conventional PCR has been used as a reliable molecular detection system for a variety of organisms in recent decades but is being replaced in many laboratories by real-time PCR analysis. Whilst the PCR method is time-consuming and methods of amplicon characterisation are laborious and not as sensitive as real-time PCR, equipment and consumable costs are much lower and therefore the technology remains more accessible to research laboratories. Additionally, conventional PCR allows amplification of much larger gene products than does real-time PCR and sequencing of products yields greater phylogenetic information. As we have shown previously, global *V. coralliilyticus* isolates contain one of two divergent Type I metalloprotease clades [18] and so classification of isolates requires that the entire *vcpA* open-reading frame be amplified by conventional PCR for meaningful sequence comparisons.

In conclusion, we successfully developed both PCR and reverse transcription real-time PCR assays for detection and quantification of the *vcpA* gene and mRNA, respectively, of *V. coralliilyticus*. While these assays do not require culturing and confirmation of individual isolates, they can obviously be used in concert with such techniques. The success of these assays in the presence of potentially interfering (and closely related) non-target DNA also suggests their use in ecological studies, where the target organism would likely be present amongst many other prokaryote and eukaryote cells. A significant result of this study is the discovery of the identical metalloproteases in *V. tubiashii* ATCC 19105 and RE22 strains causing disease in oysters and so these diagnostic tools may have important commercial applications in the aquaculture industry, as well as in reef management agencies, where early detection of virulent organisms would allow for the implementation of timely management steps to limit and possibly prevent losses due to *V. coralliilyticus* infection.

## Materials and Methods

### Bacterial strains, culture conditions and genomic DNA extraction

Global *V. coralliilyticus* isolates and strains of *Vibrio* species used in the study are listed in Table 1. Cultures of *Vibrio* species were grown overnight in Luria-Bertani broth plus 2% NaCl (LB20) on a horizontal shaker at 28°C and 200rpm. Total genomic DNA was extracted from overnight cultures using the Wizard Genomic DNA Kit (Promega, Madison, Wisconsin, USA) according to the manufacturers instructions.

### PCR primer development

The vcpAF (5-ATG AAA CAA CGT CAA ATG CTT TG-3) and vcpAR (5-CCC TTT CAC TTC CGA TGT TGT G-3) PCR primers were previously designed to amplify the entire *vcpA* open reading frame (ORF) of *V. coralliilyticus* strain P1 and subsequently used to compare the *vcpA* genes from geographically disparate isolates of *V. coralliilyticus* [18]. The Lasergene EditSeq and MegAlign software packages (DNAStar, Madison, Wisconsin, USA) were used to check that the vcpAF and vcpAR primer pairs did not match any non-target *Vibrio* species. Primer sequences were also compared with those in the NCBI database using the Nucleotide Basic Local Alignment Search Tool (BLASTn) algorithm [35]. For quantitative real-time PCR primer design, the Lasergene Primer Select software package (DNAStar) was used to analyze potential primer pairs, and their specificity and range checked *in silico* as before. The chosen primer sequences for this real-time assay were vcpARTF (5-AGC TAC GAC TGC CGC CCT TAC-3) and vcpARTR (5-GGA GCC CTT TCA CTT ACG ATG TTG-3).

### Construction of specific standard curves for Real-Time PCR

A standard curve for *V. coralliilyticus* quantification was generated by amplification of the *vcpA* ORF from *V. coralliilyticus* strain P1 genomic DNA using vcpAF forward and vcpARTR reverse primers. The amplicon was purified from agarose gels using the Nucleospin Extract II kit (Machery-Nagel, Dren, Germany), cloned into the pCR2.1-TOPO cloning vector and transformed into OneShot TOP10 competent cells as specified by the manufacturer (Invitrogen, Carlsbad, California, USA). Plasmids were prepared from transformants using the Qiagen Plasmid Purification kit (Qiagen, Valencia, California, USA) and insert size verified by agarose gel electrophoresis using SYBR Safe DNA gel stain (Invitrogen). The Lasergene SeqBuilder software package (DNAStar) was used to assess suitable restriction sites for linearisation of the plasmids such that digests did cut within the insert sequence. Plasmid DNA was quantified using a NanoDrop Spectrophotometer (Thermo Scientific, Wilmington, Delaware, USA) prior to digestion with *Bgl* II (New England Biolabs, Ipswich, Massachusetts, USA). Insert integrity was verified by PCR amplification using vcpAF and vcpARTR primers. The plasmids were serially diluted to prepare solutions containing 10^0^–10^7^ copies.

### PCR amplification

Reaction mixtures comprised 1.25 mM MgCl_2_, 200 µM dNTP, 2 U of iTaq DNA Polymerase (Biorad, Hercules, USA), 200 nM of each primer, 200 ng µL^−1^ non-acetylated BSA [71], 1 µL (2–10 ng) of genomic DNA, and nuclease-free water to bring the total volume to 50 µL. For the vcpAF and vcpAR primer pair, reactions were initially denatured for 2 min at 94°C, followed by 30 cycles of denaturation at 94°C for 1 min, primer annealing at 64°C for 1 min and extension at 72°C for 2 min 30 s. This was followed by a final extension step of 72°C for 10 min. Amplicons were visualised by agarose gel electrophoresis using SYBR Safe DNA gel stain (Invitrogen).

### RNA extraction, cDNA synthesis and real-time PCR amplification

Total RNA was extracted from early stationary phase cultures using the RiboPure Bacteria Kit (Ambion, Austin, Texas, USA). Reverse transcription was performed with the SuperScript III First Strand Synthesis System for RT-PCR (Invitrogen, Carlsbad, California, USA) as described by the manufacturer, using random hexamers to generate initial cDNA strands, which were quantified using a NanoDrop Spectrophotometer (Thermo Scientific). The real-time PCR assay comprised of 12.5 µL Qiagen Rotor-Gene SYBR Green RT-PCR Master Mix (Qiagen), 2.5 µL (10 µM) each of vcpARTF and vcpARTR primers and 2.5 µL nuclease-free water. Five microlitres of template (10 ng µL^−1^f either cDNA or plasmid DNA) was then added and each reaction performed in triplicate. A Qiagen Rotor-Gene Q real-time PCR cycler (Qiagen) was programmed with a modified Qiagen cycling program of a PCR initial activation step of 95°C for 5 min, followed by 40 cycles of denaturation of 95°C for 5 secs and a combined annealing and primer extension of 60°C for 30 secs. Quantitative PCR data was analysed using the Rotor-Gene Q software package (Qiagen).

### Sequence Analysis

PCR products were purified from agarose gels using the Nucleospin Extract II kit (Machery-Nagel) and sent to Macrogen Inc. (Seoul, Korea) for sequencing. Sequences were trimmed and contigs assembled using the Lasergene SeqMan II software package (DNAStar). The neighbour-joining method (with the ClustalW package in MegAlign) was used to construct phylogenetic trees; *V. splendidus* was included as an outgroup. Gene accession numbers for metalloprotease sequence data used for phylogeny are listed in Table S1. Sequence data for complete *vcpA* genes for *V. coralliilyticus* strains were submitted under GenBank Accession Numbers JQ345033-JQ345046.

## Supporting Information

Table S1 (TEX)
